# Beauty Re-defined: A Comparative Analysis of Artificial Intelligence-Generated Ideals and Traditional Standards

**DOI:** 10.7759/cureus.71026

**Published:** 2024-10-07

**Authors:** Isabel C Bernal, John Andre, Munir Patel, Martin I Newman

**Affiliations:** 1 College of Medicine, Dr. Kiran C. Patel College of Osteopathic Medicine, Nova Southeastern University, Fort Lauderdale, USA; 2 Plastic and Reconstructive Surgery, Cleveland Clinic Florida, Weston, USA

**Keywords:** artificial intelligence (ai), beauty standards, facial beauty, facial plastic, perception of beauty

## Abstract

Background

Traditional methods for assessing facial beauty rely on subjective measures like averages or "golden ratios." However, artificial intelligence (AI) offers a data-driven approach to analyzing attractiveness. This study explores how AI-generated beauty criteria compare to established ideals, considering cultural influences and the evolving concept of beauty.

Methods

To explore how AI-generated beauty ideals compare to traditional standards, we used three AI text-to-image generation tools (Dezgo (Dezgo SAS LLC, France), Freepik (FreePik Company, Malaga, Spain), and ImagineArt (Vyro, Islamabad, Pakistan)) to create images from a specific prompt. The first four generated images for each gender that met our criteria were included in this study. A single researcher used MediaPipe Studio software to identify ten key facial landmarks on each image. Landmark distances were measured twice in Adobe Photoshop 2023 (Adobe, San Jose, California, United States) and averaged for each measurement. The average values were then used to calculate 23 facial proportion ratios based on established neoclassical canons and golden facial ratios. We then compared these AI-generated ratios to the ideal values using one-sample t-tests in IBM SPSS Statistics for Windows, Version 29 (Released 2023; IBM Corp., Armonk, New York, United States), p < 0.05 significance, to assess alignment with traditional beauty standards.

Results

AI-generated faces displayed statistically significant differences, p < 0.05, from established beauty standards in both neoclassical canons and golden ratios for both males and females. Differences were seen in facial width, upper and lower face proportions, and eye symmetry.

Conclusion

AI-generated faces deviated from traditional beauty standards of neoclassical canons and golden ratios for both genders. This suggests AI incorporates factors beyond established ideals, potentially reflecting contemporary preferences, cultural biases, or emerging trends.

## Introduction

Facial aesthetics play a pivotal role in human attractiveness, and plastic surgeons strive for objective criteria to guide patient assessments [1]. Traditionally, "ideal" facial measurements were derived from averages of "beautiful" faces, golden ratios, or individual author preferences, often neglecting factors like age, gender, and ethnicity. However, the rise of artificial intelligence (AI) promises a dynamic shift in these approaches. For plastic surgeons, it is essential to assess and analyze patients using an objective aesthetic criterion. Literature recommendations and guidelines regarding ideal measurements for an attractive face often rely on suggested golden ratios, neoclassical canons, and “ideal” ratios and angles. The target values are typically derived from the average face perceived as “beautiful” or the author’s preferences. These values are presumed to be linked with attractive faces, irrespective of age, gender, and ethnicity.

AI, encompassing machine learning with vast datasets, has already infiltrated plastic surgery through pattern recognition techniques aiding preoperative and postoperative decisions [2,3]. This growing technology coincides with the increasing demand for objective patient evaluation methods in cosmetic procedures. Historically, defining "ideal" beauty relied on concepts like neoclassical canons and facial golden ratios. However, our perception of attractiveness is a complex tapestry woven from cultural influences, individual preferences, evolving trends, and gender nuances [4]. Recognizing this, studies have documented significant variations in facial features across ethnicities, further challenging the notion of a universal "ideal" [5]. 

This study delves into the fascinating interplay between AI-generated beauty criteria and established ideals. Given the increasing emphasis on beauty in social media, we were curious to investigate whether AI, trained on internet data, would produce images aligned with online beauty ideals or if it would reflect more diverse representations. Additionally, a literature review explores the cultural, historical, and artistic forces shaping our understanding of beauty, providing a holistic perspective on this ever-evolving landscape.

## Materials and methods

The study was conducted under the Plastic and Reconstructive Surgery Department at Cleveland Clinic Florida, Weston, Florida, United States.

Image generation and selection 

**Figure 1:**
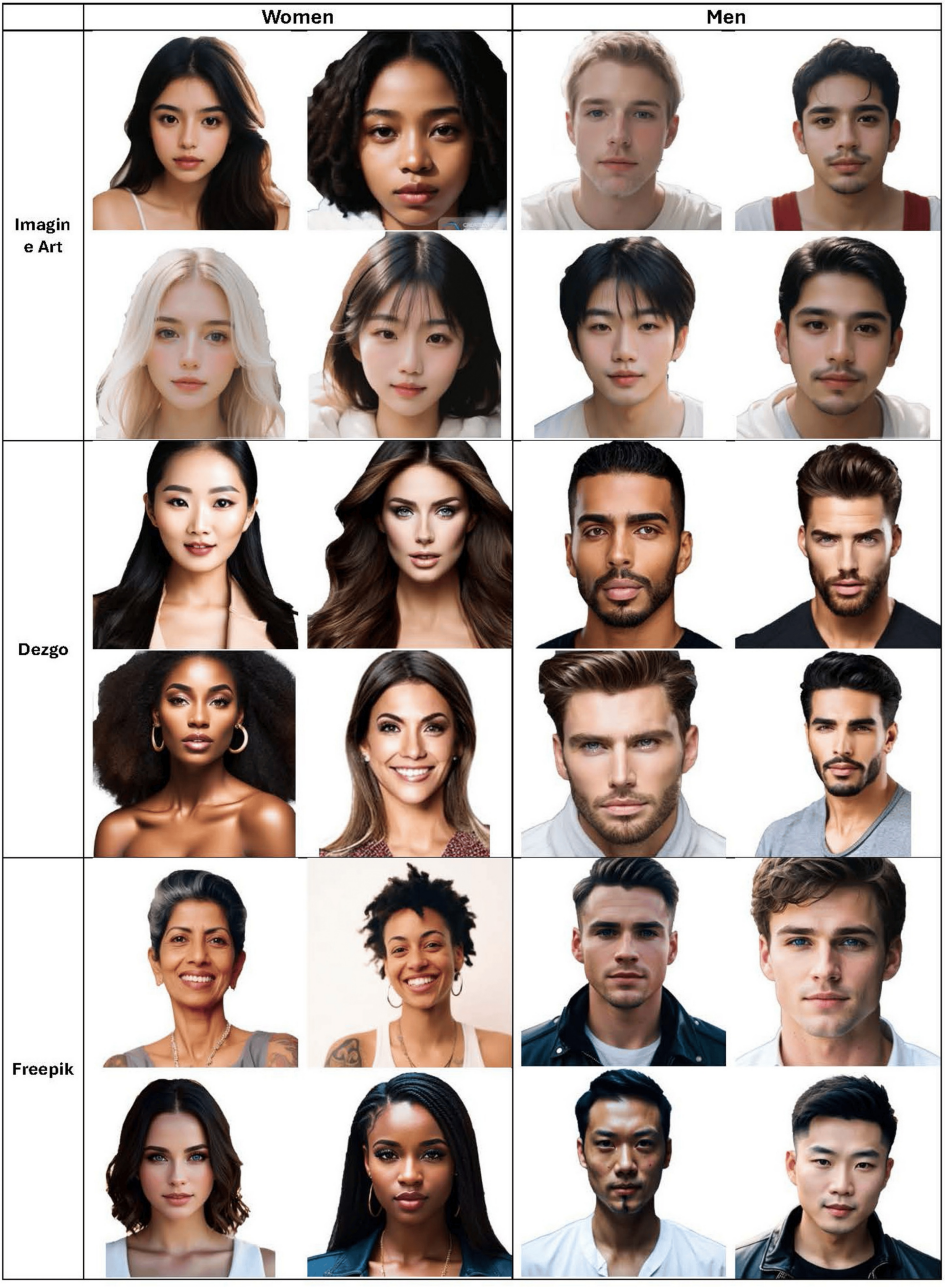
Images used for neoclassical cannon and golden ratio measurements This figure displays AI-generated images used in our study. The prompt used for a generation was a "full-face portrait of a woman/man with conventionally attractive features." AI: artificial intelligence AI text-to-image generation tools used: Dezgo (Dezgo SAS LLC, France) (https://dezgo.com/), Freepik (FreePik Company, Malaga, Spain) (https://www.freepik.com/ai/image-generator), and ImagineArt (Vyro, Islamabad, Pakistan) (https://www.imagine.art/)

This study utilized three free AI text-to-image software, Dezgo (Dezgo SAS LLC, France) [6], Freepik (FreePik Company, Malaga, Spain) [7], and ImagineArt (Vyro, Islamabad, Pakistan) [8]. Each program was queried to generate images for both men and women based on the prompt, "full-face portrait of a woman/man with the most attractive facial features." To prevent AI bias from influencing image generation, an initial search using a different platform, HotPot AI (Panabee, LLC, Palo Alto, California, United States) was conducted to refine the query. Subsequently, a single researcher generated the first four images for each gender that met our criteria on each of the target platforms, resulting in a dataset of 24 images (12 male, 12 female) (Figure 1).

Image selection was based on specific criteria: neutral facial orientation, accurate anatomical features, and clear visibility of all ten facial landmarks. To standardize image appearance and emphasize facial features, all images were processed in Adobe Photoshop 2023 (Adobe, San Jose, California, United States) to remove backgrounds, resize, and pixelate them, and saved them as JPG files.

Landmark identification and data extraction

Ten key facial landmarks, detailed in Table 1, were identified on each image by a single researcher using MediaPipe Studio facial recognition software. To enhance measurement consistency and address the challenges posed by manual methods and image variability, we adopted a standardized approach. By utilizing MediaPipe Studio (Google LLC, Mountain View, California, United States) to identify specific facial landmarks, we established consistent reference points for each measurement. To minimize measurement error, a single researcher independently measured the distances between these landmarks twice in Adobe Photoshop 2023. The average of these measurements was then used to calculate facial ratios based on established neoclassical canons and golden ratios.

**Table 1:** Anatomic facial landmarks This table provides the definitions and abbreviations of the facial landmarks used in this study.

Landmark Abbreviations	Anatomic Explanation
Trichion (Tr)	The highest point is in the midline of the hairline, directly above the forehead.
Exocanthion (Ex)	The outermost point of the eye is where the upper and lower eyelids meet.
Endocanthion (En)	The innermost point of the eye is where the upper and lower eyelids meet.
Nasion (N)	The deepest point at the bridge of the nose, in line with the upper lash line.
Subnasale (Sn)	The lowest point is at the base of the columella directly, above the upper lip.
Ala (Al)	The most lateral point is on the outer edge of the alar wings of the nose.
Cheilion (Ch)	The corner of the mouth where the upper and lower lip meet.
Zygion (Zy)	The most prominent point is on the cheekbone.
Menton (Me)	The most anterior inferior point on the chin.
Gnathion (Gn)	The most inferior point of the midline of the lower jaw.

Comparison with established ideals

**Figure 2:**
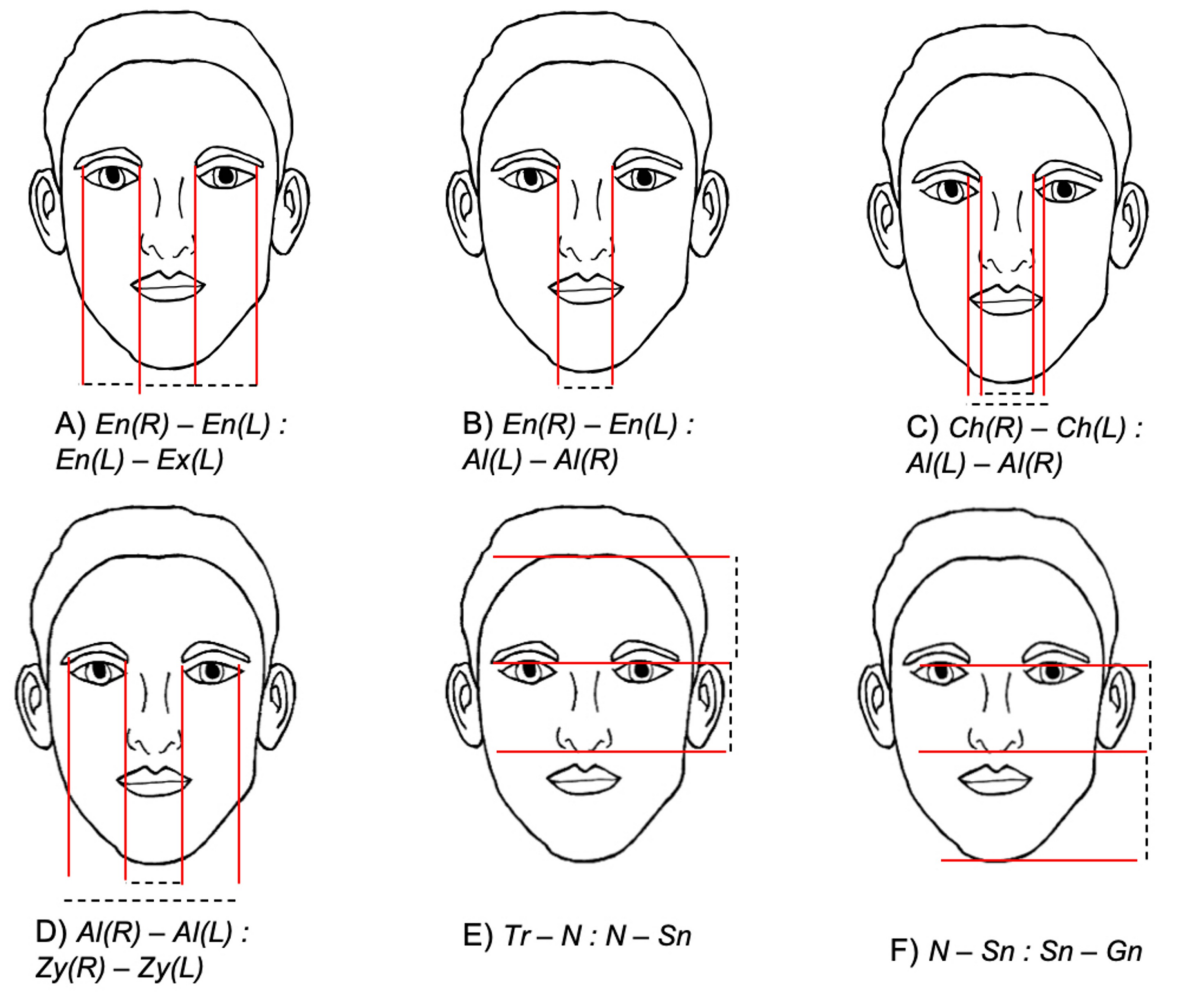
Graphical representation of the six neoclassical canon ratios used in this study This figure illustrates the neoclassical canons used for calculating ratios analyzed in this study. Dotted lines depict the specific distances measured between landmarks to derive each ratio. A)* Orbital canon (Right and Left)*: intercanthal distance 'Endocanthion(*En)-*Endocanthion*(En)'* equals the width of the eye 'Endocanthion(*En)-*Exocanthion*(Ex*)'; B)* Orbitonasal canon*: intercanthal distance 'Endocanthion​​​​​​​(*En*)-Endocanthion(*En*)' equals the nasal width 'Ala(*Al*)-Ala(*Al*)'; ​​​​​​​C)* Naso-oral canon*: mouth width 'Cheilion​​​​​​​(*Ch)-*Cheilion​​​​​​​(*Ch*)' is equal to 1.5 widths of the nose 'Ala(*Al*)-Ala(*Al*)'; ​​​​​​​D)* Nasofacial canon*: where the nose width 'Ala(*Al*)-Ala(*Al*)' is equal to one-quarter of the face width 'Zygion(*Zy)-*Zygion*(Zy*)'; ​​​​​​​E)* Upper to middle facial third*: trachion to nasion 'Trichion(*Tr)-*Nasion*(N*)' compared to nasion to subnasale 'Nasion(*N)-*Subnasale*(Sn*)'; ​​​​​​​F)* Middle to lower facial third*: nasion to subnasale 'Nasion(*N)-*Subnasale*(Sn*)' compared to subnasale to gnathion 'Subnasale(*Sn)-*Gnathion(*Gn*)'

**Figure 3:**
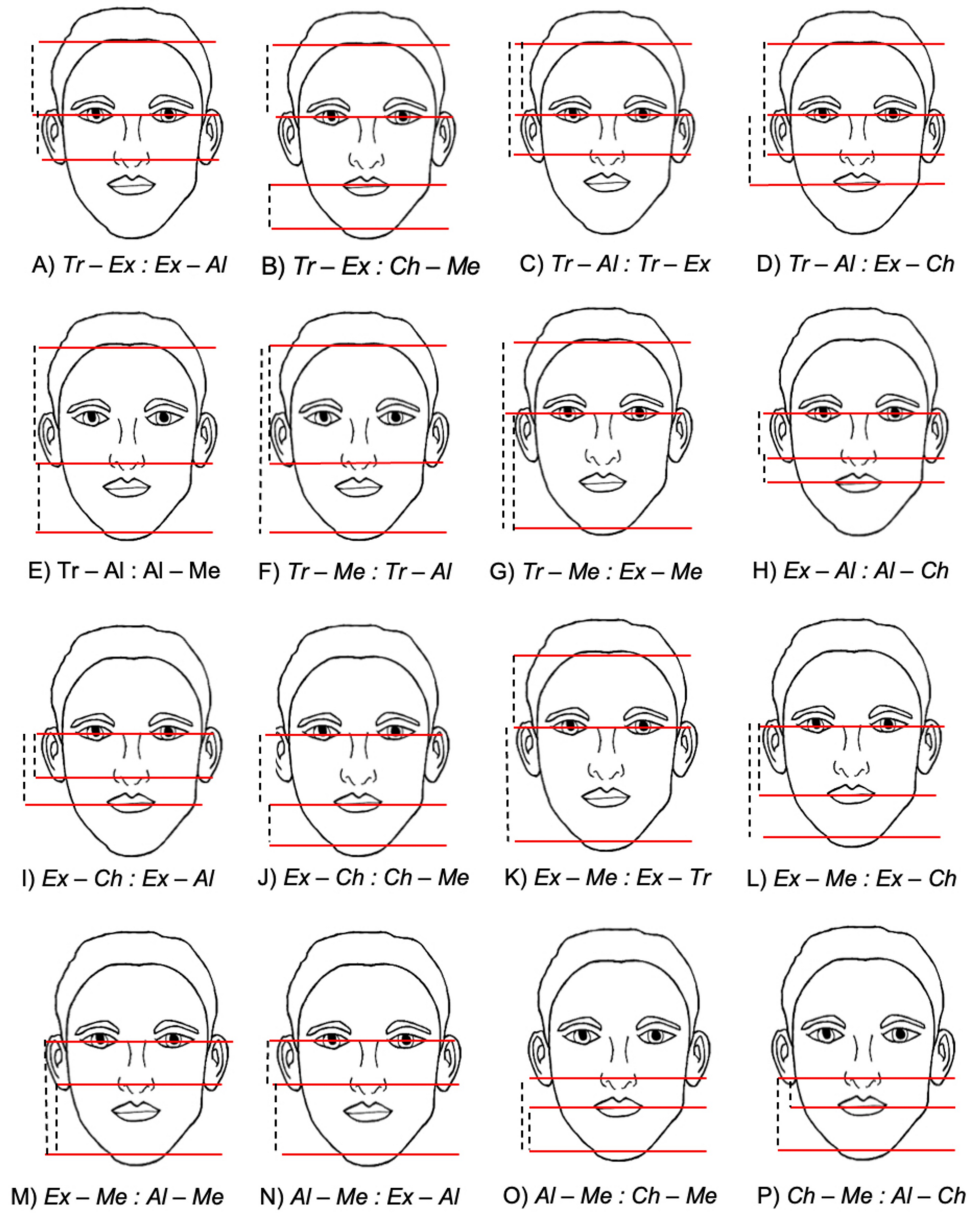
Graphical representation of the golden ratios used in this study This figure illustrates the golden ratios (labeled) used to calculate the ratios analyzed in this study. Dotted lines depict the specific distances measured between landmarks to derive each ratio. A) Trichion*(Tr)*-Exocanthion(*Ex)* : Exocanthion*(Ex)*-Ala*(Al); *B) Trichion*(Tr)*-Exocanthion*(Ex) *: Cheilion*(Ch)*-Menton*(Me); *C) Trichion*(Tr)*-Ala*(Al) *: Trichion*(Tr)*-Exocanthion*(Ex); *D) Trichion*(Tr)*-Ala*(Al) *: Exocanthion*(Ex)*-Cheilion*(Ch); *E) Trichion*(Tr)*-Nasion*(N) *: Nasion*(N)*-Menton*(Me); *F) Trichion*(Tr)*-Menton*(Me)* : Trichion*(Tr)*-Ala*(Al); *G) Trichion*(Tr)*-Menton*(Me)* : Exocanthion*(Ex)*-Menton*(Me); *H) Exocanthion*(Ex)*-Nasion*(N)* : Nasion*(N)*-Cheilion*(Ch); *I) Exocanthion*(Ex)*-Cheilion*(Ch) *: Exocanthion*(Ex)*-Ala*(Al); *J) Exocanthion*(Ex)*-Cheilion*(Ch) *: Cheilion*(Ch)*-Menton*(Me); K) *Exocanthion*(Ex)*-Menton*(Me)* : Exocanthion*(Ex)*-Trichion*(Tr); L) *Exocanthion*(Ex)*-Menton*(Me)* : Exocanthion*(Ex)*-Cheilion*(Ch); M)*Exocanthion*(Ex)*-Menton*(Me)* : Ala*(Al)*-Menton*(Me); *N) Ala*(Al)*-Menton*(Me)* : Exocanthion*(Ex)*-Ala*(Al); *O) Ala*(Al)*-Menton*(Me)* :  Cheilion*(Ch)*-Menton*(Me); *P) Cheilion*(Ch)*-Menton*(Me)* : Ala*(Al)*-Cheilion*(Ch)*

We calculated 23 facial proportion ratios based on established beauty standards, six neoclassical canons (Figure 2), and 16 golden facial ratios (Figure 3) for each image across genders and AI programs. For each gender, we calculated the average value across the four images for each ratio. These average ratios represent the typical AI-generated facial proportions for each ideal. We then compared these average ratios to the ideal ratio value using one-sample t-tests. This comparison allowed us to assess whether AI-generated proportions significantly differed from the traditional beauty standards.

Statistical analysis

For each gender and AI program, we calculated the average of the measurements of each individual ratio across all four generated images. These resulting mean ratios represented the average facial proportion for each standard ideal proportion per AI software program within a specific gender group. We then compared our values to the established ideals derived from neoclassical canons and golden facial ratios using one-sample t-tests in IBM SPSS Statistics for Windows, Version 29 (Released 2023; IBM Corp., Armonk, New York, United States). Statistical significance was set at a p-value threshold of p < 0.05.

## Results

We asked three AI models to generate portraits with varying facial features reflecting their interpretations of "ideal beauty." Using the previously established beauty ideals of six neoclassical canons and 16 golden facial ratios, we calculated 23 mean facial proportion values for each gender. Our findings were then compared to the ideal values of each established ideal. Male portraits revealed statistically significant deviations from two neoclassical cannons, primarily related to facial width and the upper two-thirds of the face. Females exhibited similar deviations, with four out of 23 ratios showing significant differences in eye symmetry, facial width, and the upper two-thirds of the face. Analysis of golden facial ratios identified a few significant differences for both genders. Males exhibited differences in two ratios of the lower face, while females presented with three ratios concentrated in the mid and lower face. Tables 2, 3 present all of our findings. 

**Table 2:** P-values of neoclassical canons compared to established ideals This table presents p-values for the neoclassical canons for each gender of AI-generated images compared to their respective standard ideal value. * indicates p < 0.05. Tr: Trichion; Ex: Exocanthion; Al: Ala; Ch: Cheilion; En: Endocanthion' Zy: Zygion; N: Nasion; Sn: Subnasale

Neoclassical Canons		En(R)-En(L): En(L)-EX(L)	En(R)-En(L): En(R)-Ex(R)	En(R)-En(L): Al(R)-Al(L)	Ch(R)-Ch(L): Al(R)-Al(L)	Al(R)-Al(L): Zy(R)-Zy(L)	Tr-N: N-Sn	N-Sn: Sn-Gn
Ideal Values		1	1	1	1.5	0.25	1	1
Male Mean	Imagine Art	1.002	1.034	0.808	1.414	0.36	1.291	0.814
	FreePik	1.058	1.093	0.938	1.531	0.33	1.212	1.022
	Dezgo	1.05	1.128	0.871	1.314	0.36	1.178	0.908
	p-value	1.74	0.076	0.9	0.329	0.006*	0.021*	0.29
Female Mean	Imagine Art	1.056	1.146	0.964	1.502	0.33	1.429	0.994
	FreePik	1.125	1.192	0.994	1.528	0.33	1.43	0.889
	Dezgo	1.097	1.176	0.934	1.439	0.35	1.443	0.987
	p-value	0.044*	0.006*	0.174	0.735	0.008*	< .001*	0.328

**Table 3:** P-values of facial ratios compared to the golden ratio This table presents p-values for the facial ratios of each gender of AI-generated images compared to the golden ratio of 1.618 * indicates p < 0.05. Tr: Trichion; Ex: Exocanthion; Ch: Cheilion; Me: Menton; Al: Ala

Facial Ratios		Tr-Ex′: Ex′-Al′	Tr-Ex′: Ch′-Me	Tr-Al′: Tr-Ex′	Tr-Al′: Ex′-Ch′	Tr-Al′: Al′-Me	Tr-Me: Tr-Al′	Tr-Me: Ex′-Me	Ex′-Al′: Al-Ch′	Ex′-Ch′: Ex′-Al′	Ex′-Ch′: Ch′-Me	Ex-Me′: Ex′-Tr	Ex′-Me: Ex′-Ch′	Ex′-Me: Al′-Me	Al′-Me: Ex′-Al′	Al′-Me: Ch′-Me	Ch′-Me: Al′-Ch′
Ideal Values		1.618	1.618	1.618	1.618	1.62	1.618	1.618	1.618	1.618	1.618	1.618	1.618	1.618	1.618	1.618	1.618
Male Mean	Imagine Art	1.804	1.344	1.512	1.535	1.25	1.817	1.583	1.029	1.772	1.319	1.732	1.919	1.423	2.217	1.634	1.327
	FreePik	1.981	1.685	1.505	1.708	2.54	1.671	1.696	1.262	1.739	1.481	1.487	1.692	2.139	1.585	1.377	1.485
	Dezgo	1.689	1.419	1.596	1.477	1.76	1.745	1.556	1.002	1.821	1.536	1.795	1.661	1.969	2.003	1.262	1.199
	p-value	0.135	0.32	0.11	0.586	0.6	0.096	0.895	0.024*	0.022*	0.118	0.626	0.229	0.405	0.23	0.22	0.077
Female Mean	Imagine Art	1.84	2.017	1.481	1.777	1.71	1.705	2.044	1.434	1.617	1.682	1.399	1.45	1.39	1.684	1.756	1.361
	FreePik	2.155	1.821	1.471	1.75	2.98	1.628	1.994	1.107	1.857	1.561	1.038	1.49	2.54	1.184	1.007	1.314
	Dezgo	2.199	2.024	1.46	1.757	2.22	1.602	1.75	1.148	1.827	1.688	1.339	1.609	2.053	1.672	1.563	1.251
	p-value	0.59	0.37	0.002*	0.003*	0.21	0.473	0.076	0.063	0.187	0.596	0.085	0.167	0.376	0.589	0.515	0.01*

## Discussion

This study investigated the relationship between AI-generated beauty criteria and established traditional ideals in the literature. Our analysis, revealing significant deviations from neoclassical canons and golden facial ratios, suggests that AI models may interpret "ideal beauty" through a lens broader than the long-standing standards. While this finding acknowledges the continued influence of traditional ideals, it also highlights the potential incorporation of contemporary aesthetic preferences, cultural biases within training data, and even emerging trends. This dynamic interplay between AI and established ideals raises several intriguing implications. AI's adaptability reflects and potentially accelerates the evolution of beauty standards, suggesting a future where established ideals are challenged and redefined. Understanding how AI incorporates diverse factors can inform plastic surgeons, encouraging a move beyond standardized ideals toward personalized approaches that respect cultural nuances and individual preferences.

The historical journey of beauty ideals paints a fascinating picture of constant evolution. While ancient civilizations linked beauty to moral virtues, the Renaissance ushered in an era of anatomical study and idealized proportions championed by figures like Leonardo da Vinci. Fast forward to the 20th century, and plastic surgery embraced facial symmetry and proportion [9]. However, the mid-20th century witnessed a shift as Hollywood stars like Marilyn Monroe and Audrey Hepburn became cultural icons, shaping beauty standards [10]. Plastic surgery literature during this era reflected this convergence towards standardized, "silver screen" ideals.

Beyond proportions and symmetry, contemporary literature introduces the vital concept of facial harmony, and the overall coherence of features for aesthetic appeal [11]. Furthermore, cultural nuances and patient-centricity have diversified beauty ideals, recognizing the influence of diverse cultural aesthetics [12]. Recent decades have brought a paradigm shift. Patient-centric approaches emphasizing personalized enhancements and acknowledging diverse cultural aesthetics have gained traction [13]. This shift aligns beautifully with the adaptability seen in AI models, which showcase a broader diversity reflecting evolving societal preferences [14].

This study recognizes the inherently subjective nature of beauty and the limitations associated with its quantification. Additionally, we acknowledge the potential ethical concerns surrounding AI's impact on individuals' self-esteem and mental well-being. These are critical considerations that demand careful attention as we explore the intersection of AI, cultural aesthetics, and the complex history of beauty ideals in plastic surgery.

While our research opens numerous avenues for future exploration, several key areas present compelling opportunities for advancement. Firstly, investigating the incorporation of additional objective criteria, such as facial harmony and skin texture, into AI models could not only enhance their accuracy but also expand their applicability. However, delving deeper is crucial. Further research is needed to pinpoint the specific factors influencing AI's beauty criteria, allowing us to mitigate potential biases and ensure inclusivity within these models effectively. Finally, we must investigate the broader societal impact of AI-shaped beauty perceptions on self-esteem and well-being, particularly among vulnerable populations.

Limitations

This study acknowledges the inherent subjectivity of beauty and the potential for cultural bias to influence our analysis. Relying on established aesthetic standards, which themselves are shaped by cultural norms, carries the risk of perpetuating existing biases. Additionally, the AI models used may incorporate biases reflecting the demographics of their training data, as seen in facial recognition algorithms [15]. While the adaptability of AI holds promise, mitigating these potential biases is crucial to ensuring ethical and inclusive applications in defining beauty standards. Therefore, our findings should be interpreted with caution. Recognizing the limitations imposed by subjectivity and cultural influences guides the need for further research. One of the AI software's most intriguing features is its capacity to learn from each search. However, a limitation of our study was the need for multiple images from the same software. This necessitated repeated input of the same term. While this could allow the AI to learn from previous searches, it also provides a more accurate representation of a population's diverse facial features and beauty. Addressing these limitations through future research will ensure a more comprehensive and responsible exploration of the complex relationship between AI, cultural aesthetics, and the ever-evolving landscape of beauty.

## Conclusions

Our analysis revealed that AI-generated faces deviated from established beauty standards, represented by both neoclassical canons and golden facial ratios, in a significant number of cases. This suggests that, while traditional ideals still hold some influence, AI models incorporate additional factors when interpreting "ideal beauty." These factors could encompass contemporary aesthetic preferences, cultural biases present in training data, or even emerging trends reflected in media. Deviations were observed in specific features like facial width in both men and women, while men additionally showed differences in the upper two-thirds and women in the mid-face compared to standard beauty ideals. This highlights the dynamic interplay between AI and traditional beauty standards, with the potential for AI to reshape our perceptions over time. Further investigation into the specific drivers of these deviations and their wider societal implications would be valuable to understanding the evolving landscape of beauty in the age of artificial intelligence.
